# Association between sedentary behavior, screen time and metabolic syndrome among Chinese children and adolescents

**DOI:** 10.1186/s12889-024-19227-w

**Published:** 2024-06-27

**Authors:** Xue Cheng, Qiya Guo, Lahong Ju, Weiyi Gong, Xiaoqi Wei, Xiaoli Xu, Liyun Zhao, Hongyun Fang

**Affiliations:** 1 National Institute for Nutrition and Health, Chinese Center for Disease Control and Prevention, Beijing, 100050 China; 2 NHC Key Laboratory of Trace Element Nutrition, National Institute for Nutrition and Health, Chinese Center for Disease Control and Prevention, Beijing, 100050 China

**Keywords:** Sedentary time, Screen time, Metabolic syndrome, Children and adolescents, China

## Abstract

**Background:**

The aim of the study was to investigate the relationship between sedentary behavior, screen time and MetS among Chinese children and adolescents aged 7–17 years. Data was obtained from the China National Nutrition and Health Surveillance of Children and Lactating Mothers in 2016–2017.

**Methods:**

Data on sedentary time, screen time, and MetS indicators were obtained through physical and health questionnaires, anthropometric measurements, and clinical examinations. MetS was defined according to the Cook’s criteria. Wilcoxon rank sum test and chi-square test were applied for comparisons of measurement data and counting data, respectively. The relationship between sedentary time, screen time, and MetS and its components was analyzed using a multivariate logistic regression model.

**Results:**

The prevalence of MetS among 7-17-year-old students in 2016–2017 was 5.45%. Compared to those with low sedentary behavior, in high sedentary behavior groups, the prevalence of abdominal obesity, high TG, low HDL-C, and MetS was high in boys, and the prevalence of abdominal obesity, high TG, hyperglycemia, and MetS was high in girls. Moreover, for those who reported ≥ 3 h/day of screen time, the prevalence of abdominal obesity, low HDL-C, and MetS was higher in boys, and the prevalence of abdominal obesity and MetS was higher in girls. After adjusting for confounding variables, the risks of abdominal obesity, high TG, low HDL-C, and MetS were higher in high-level sedentary time group, and the risks of abdominal obesity and MetS were 1.15 and 1.14 times higher for those who spent ≥ 3 h/day on screen time, respectively.

**Conclusions:**

This study shows that high levels of sedentary time and screen time were associated with an increased likelihood of MetS among Chinese children and adolescents aged 7–17 years. Reducing sedentary behavior and screen time may contribute to the prevention of metabolic diseases.

## Background

Metabolic syndrome (MetS), a cluster of interconnected factors that directly increase the risk of cardiovascular disease (CVD) and type 2 diabetes mellitus (T2DM) [1], has drawn public attention for decades. In recent years, the prevalence of MetS in children and adolescents has gradually increased and has became a worldwide public health issue. In 2020, 2.8% children and 4.8% adolescents had MetS globally, equating to approximately 25.8 million children and 35.5 million adolescents [2]. According to Noubiap et al., among the 44 countries in their study, Nicaragua (5.2%), Iran (8.8%), and Mexico (12.3%) had the highest prevalence of MetS in children, and Iran (9.0%), United Arab Emirates (9.8%), and Spain (9.9%) had the highest prevalence of MetS in adolescents [2].

Sedentary behavior refers to any waking behavior such as sitting or learning with an energy expenditure of 1.5 metabolic equivalent task or less [3]. A sedentary lifestyle increases all-cause mortality and the risks for CVD, diabetes mellitus (MD), hypertension (HTN), and cancers [4, 5]. Nowadays, sedentary time remains high among children and adolescents, and in many countries, the trend keeps rising over the past few decades. Across the 97 countries with knowledge on sitting in the Global School-based Student Health Survey (GSHS), 25% boys and 24% girls aged 13–15 years reported sitting for more than 3 h/day in addition to sitting at school and for homework [6]. From 2007 to 2016, sitting time of U.S. adolescents increased from 7 h per day to 8.2 h per day [7].

A growing body of literature suggests that excessive use of electronic devices could lead to poor eye health [8, 9], risks of chronic disease such as obesity [10, 11], hypertension [12, 13], and insulin resistance [14], poor sleep [9], depression and anxiety [15, 16]. 95% U.S. adolescents reported having or accessing to a smartphone [17]. Prevalence of 11-15-year-olds watching TV for ≥2 h on weekdays ranged from 45% (Switzerland) to 69% (United Kingdom, Wales) among boys, and from 40% (Switzerland) to 72% (Bulgaria) among girls. Prevalence of playing computer games for ≥2 h on weekdays ranged from 32% (Switzerland) to 68% (Denmark) in boys, and from 11% (Finland) to 47% (Netherlands) in girls [6].

Studies have revealed that sedentary behavior and high screen time are associated with an increased likelihood of MetS [18–20], while modification of lifestyle-associated risk factors is critical for the prevention and management of MetS [21]. Meanwhile, childhood and adolescence are critical periods for establishing healthy living habits [22]. Therefore, it is necessary to assess and manage risk factors of MetS to improve their lives. We used the data from the China National Nutrition and Health Surveillance of Children and Lactating Mothers in 2016–2017 to investigate the relationship between sedentary behavior, screen time and MetS among Chinese children and adolescents aged 7–17 years.

## Methods

### Study design and participants

Data was obtained from the China National Nutrition and Health Surveillance of Children and Lactating Mothers in 2016–2017 [23]. A complex, stratified multi-stage cluster random sampling design was performed. 31 provinces in China were divided into four categories: large cities, small and medium-sized cities, rural areas, and impoverished rural areas. Among these four types of areas, 275 survey sites were randomly selected, including 31 large cities, 101 small and medium-sized cities, 97 rural areas, and 46 impoverished rural areas. At each survey site, two primary schools, two junior high schools, and one senior high school were randomly selected, and one class was selected from each grade. Grade 9 and grade 12 were excluded because of the poor compliance. A total of 280 students were chosen from each survey site, with equal number of boys and girls. Participants with major physical and mental illness were excluded.

The study was approved by the ethical committee of the National Institute for Nutrition and Health of Chinese Center for Disease Control and Prevention (approval No.: 201,614). The informed consent was signed by all participants or their guardians.

### Survey methods and contents

Four parts were created for and included in the China National Nutrition and Health Surveillance of Children and Lactating Mothers in 2016–2017 [23], and three of them were used in this study: physical and health questionnaires, anthropometric measurements, and clinical examinations. Basic personal and family information, lifestyle, health condition, and physical activity were included in the questionnaire. The questionnaire was evaluated and verified by experts from Chinese Center for Disease Control and Prevention and has been used for several rounds of the China National Nutrition and Health Surveillance. Referring to the Global Physical Activity Questionnaire from WHO and combining with the national condition of China, the following questionnaire items on sedentary behaviors was included: (1) study; (2) watching TV and videos; (3) using cell phones; (4) playing video games; (5) playing tablets; (6) using other electronic devices; and (7) reading while sitting or reclining. In this study, item (1) to (7) were used to calculate the sedentary time, and item (2) to (6) were used to calculate the screen time. All questionnaires were asked and completed face-to-face by uniformly trained investigators.

All questionnaire data were collected and entered in the national system platform by trained CDC employees. Extreme values were excluded. Data with no personal information (id, age, sex, etc.) and no screen time and sedentary time values were excluded. For item (6) using other electronic devices, duplicate values and activities with other items were deleted. Answers of item (1) to (7) such as “none” “no” and “nothing else” were set as value 0.

### Anthropometric measurements and clinical examinations

Waist circumference (WC) was measured horizontally at the midpoint between the inferior edge of the costal arch and the iliac crest in the midaxillary line using a tape. Systolic blood pressure (SBP) and diastolic blood pressure (DBP) were measured three times in one-minute intervals by an electronic sphygmomanometer (Omron HBP 1300, Tokyo, Japan). The mean value of the two nearest measurements were calculated.

6 mL fasting blood samples were collected for triglyceride (TG), high-density lipoprotein cholesterol (HDL-C), and fasting blood glucose (FBG). FBG was measured using glucokinase method (Roche P800 automatic biochemical analyzer), and TG and HDL-C were measured using enzyme colorimetry (Roche Cobas C701 automatic biochemical analyzer series).

### Metabolic syndrome

MetS was defined according to the Cook’s criteria [24], a modified version of the National Cholesterol Education Program-Adult Treatment Panel III (NCEP-ATP III) criteria for children and adolescents. Participants having at least 3 of the following 5 risk factors were defined as having MetS: (1) abdominal obesity: WC≥ 90th percentile for age and sex, determined by the cutoff points for Chinese children and adolescents [25], (2) elevated blood pressure: SBP or DBP≥ 90th percentile for age, sex, and height [26], (3) high triglycerides: serum TG≥ 1.24 mmol/L, (4) low HDL-C: HDL-C≤ 1.03 mmol/L, (5) high fasting blood glucose: FBG≥ 6.1 mmol/L.

### Diagnostic criteria

The sedentary time was categorized in quartiles. And based on the data and its distribution, three levels of sedentary time were made: low level (Q1): < 6.26 h/day, medium level (Q1-Q3): 6.26–9.75 h/day, and high level(Q3): >9.75 h/day. There were 14,624, 29,406, and 14,682 participants in the low-level, medium-level and high-level groups, respectively. The screen time was categorized into three groups according to the Canadian Sedentary Behaviour Guidelines for Children and Youth [27] and the distribution of data: < 2 h/day, 2–3 h/day and ≥ 3 h/day.

### Statistical analysis

SAS software (v.9.4, SAS Institute Inc., Cary, NC, USA) was applied. For non-normal distribution data, median and interquartile range (IQR) were used to describe continuous variables; and categorical variables were reported as frequency (percentage). Wilcoxon rank sum test was applied to compare measurement data between groups, and chi-square test was used for comparison of counting data. The relationship between sedentary time, screen time, and MetS and its components was analyzed using a multivariate logistic regression model, and the effect of confounding factors were adjusted. A two-sided *p* < 0.05 was considered statistically significant.

## Results

A total of 58,712 participants were included in the study. The prevalence of MetS among 7-17-year-old students in 2016–2017 was 5.45%. The prevalence of abdominal obesity, high TG, low HDL-C, hyperglycemia, and HTN were 15.58%, 15.92%, 11.34%, 1.63%, 38.14%, respectively. Boys had higher prevalence of MetS than girls (*p* < 0.05). With an increasing age, the prevalence of MetS increased (*p* < 0.05). In addition, urban students were more likely to have MetS than rural students; and high levels of sedentary time and screen time had higher prevalence of MetS (*p* < 0.05). In terms of MetS components, all of them had worse profiles in MetS group than those in non-MetS group (*p* < 0.05) (Table 1).

As shown in Table 2, in boys, the prevalence of abdominal obesity, high TG, low HDL-C, and MetS increased with an increase in sedentary time (*p* < 0.0001). In girls, the prevalence of abdominal obesity, high TG, hyperglycemia, and MetS was higher in high-level sedentary time group than those in low-level sedentary time group (*p* < 0.05). As far as screen time, in boys, the prevalence of abdominal obesity, low HDL-C, and MetS increased with the increase in screen time (*p* < 0.05); and in girls, the prevalence of abdominal obesity and MetS was higher for those who spent ≥ 3 h/day on screen time (*p* < 0.05) (Table 3).

**Table 1:** Demographic characteristics according to MetS status

Variables	MetS	Non-MetS	Total
Gender*			
Boy	1664 (5.67)	27,700 (94.33)	29,364 (50.01)
Girl	1538 (5.24)	27,810 (94.76)	29,348 (49.99)
School*			
Primary	1788 (4.88)	34,832 (95.12)	36,620 (62.37)
Junior high	866 (6.39)	12,682 (93.61)	13,548 (23.08)
Senior high	548 (6.41)	7996 (93.59)	8544 (14.55)
Residential area*			
Urban	1736 (6.22)	26,183 (93.78)	27,919 (47.55)
Rural	1466 (4.76)	29,327 (95.24)	30,793 (52.45)
Per capita household income (CNY/year)			
< 30,000	196 (5.12)	3630 (94.88)	3826 (6.52)
30,000-50000	257 (5.58)	4349 (94.42)	4606 (7.85)
50,000-80000	320 (5.76)	5235 (94.24)	5555 (9.46)
≥ 80,000	293 (5.59)	4951 (94.41)	5244 (8.93)
N/A	2136 (5.41)	37,345 (94.59)	39,481 (67.25)
Average sedentary time (hours/day)*	8.29 (3.71)	7.79 (3.48)	7.80 (3.49)
Average screen time (hours/day)*	1.38 (1.71)	1.29 (1.64)	1.29 (1.64)
Sedentary time level*			
Low	622 (4.25)	14,002 (95.75)	14,624 (24.91)
medium	1612 (5.48)	27,794 (94.52)	29,406 (50.09)
high	968 (6.59)	13,714 (93.41)	14,682 (25.01)
Screen time (hours/day)*			
< 2	2105 (5.23)	38,170 (94.77)	40,275 (68.60)
2–3	518 (5.50)	8895 (94.50)	9413 (16.03)
≥ 3	579 (6.42)	8445 (93.58)	9024 (15.37)
Physical Activity(hours/day)			
≤ 60	1248 (5.26)	22,481 (94.74)	23,729 (40.42)
61–120	833 (5.75)	13,655 (94.25)	14,488 (24.68)
> 120	1121 (5.47)	19,374 (94.53)	20,495 (34.90)
Waist circumference (cm)*	79.85 (15.85)	61.90 (13.25)	62.35 (14.06)
Serum triglyceride (mmol/L)*	1.52 (0.62)	0.79 (0.40)	0.81 (0.44)
Serum HDL-C (mmol/L)*	1.00 (0.29)	1.40 (0.42)	1.39 (0.44)
Fasting glucose (mmol/L)*	5.07 (0.72)	5.00 (0.66)	5.00 (0.66)
Systolic blood pressure (mmHg)*	123.33 (13.33)	110.33 (15.00)	111.00 (15.67)
Diastolic blood pressure (mmHg)*	71.67 (11.00)	65.33 (11.00)	65.67 (11.00)
ALL	3202 (5.45)	55,510 (94.55)	58,712

Data was presented as median (IQR) or as frequency values (percentage). *: *p*-values were obtained by chi-square test or Wilcoxon rank sum test, *p* < 0.05

**Table 2:** Prevalence of MetS components in different sedentary time groups

	Boys (*n* = 29,364 )				Girls (*n* = 29,348 )			
Variables	Low level *n* (%)	Medium level *n* (%)	High level *n* (%)	*p*-Value	Low level *n* (%)	Medium level *n* (%)	High level *n* (%)	*p*-Value
Abdominal obesity	935 (12.89)	2246 (15.16)	1446 (19.81)	< 0.0001	913 (12.38)	2270 (15.56)	1337 (18.11)	< 0.0001
High TG	948 (13.07)	2005 (13.54)	1154 (15.81)	< 0.0001	1203 (16.32)	2687 (18.41)	1350 (18.29)	0.0004
Low HDL-C	617 (8.51)	1712 (11.56)	1206 (16.52)	< 0.0001	754 (10.23)	1581 (10.83)	790 (10.70)	0.3831
Hyperglycemia	144 (1.99)	289 (1.95)	170 (2.33)	0.1576	71 (0.96)	180 (1.23)	105 (1.42)	0.0371
Hypertension	2847 (39.26)	5530 (37.33)	2688 (36.83)	0.0047	2971 (40.30)	5714 (39.16)	2644 (35.81)	< 0.0001
Number of MetS components				< 0.0001				< 0.0001
0	3311 (45.66)	6730 (45.43)	3067 (42.02)		3142 (42.62)	6108 (41.86)	3160 (42.80)	
1	2755 (37.99)	5338 (36.04)	2524 (34.58)		2907 (39.43)	5507 (37.74)	2701 (36.58)	
2	870 (12.00)	1965 (13.27)	1140 (15.62)		1017 (13.80)	2146 (14.71)	1122 (15.20)	
≥ 3 (MetS)	316 (4.36)	780 (5.27)	568 (7.78)		306 (4.15)	832 (5.70)	400 (5.42)	
Total	7252 (24.70)	14,813 (50.45)	7299 (24.86)		7372 (25.12)	14,593 (49.72)	7383 (25.16)	

values were obtained by chi-square test

**Table 3:** Prevalence of MetS components in different screen time groups

	Boys (*n* = 29,364)				Girls (*n* = 29,348)			
Variables	< 2 h/day *n* (%)	2–3 h/day *n* (%)	≥ 3 h/day *n* (%)	*p*-Value	< 2 h/day *n* (%)	2–3 h/day *n* (%)	≥ 3 h/day *n* (%)	*p*-Value
Abdominal obesity	2977 (15.37)	772 (15.56)	878 (17.46)	0.0013	3070 (14.69)	708 (15.91)	742 (18.58)	< 0.0001
High triglyceride	2666 (13.76)	683 (13.76)	758 (15.07)	0.0519	3742 (17.90)	749 (16.83)	749 (18.75)	0.0662
Low HDL-C	2237 (11.55)	575 (11.59)	723 (14.37)	< 0.0001	2244 (10.74)	451 (10.13)	430 (10.77)	0.4798
Hyperglycemia	402 (2.08)	97 (1.95)	104 (2.07)	0.8649	245 (1.17)	53 (1.19)	58 (1.45)	0.3300
Hypertension	7379 (38.09)	1850 (37.28)	1836 (36.50)	0.0952	8172 (39.09)	1733 (38.94)	1424 (35.65)	0.0002
Number of MetS components				< 0.0001				0.0009
0	8591 (44.35)	2291 (46.17)	2226 (44.25)		8760 (41.91)	1933 (43.43)	1717 (42.99)	
1	7170 (37.01)	1709 (34.44)	1738 (34.55)		8025 (38.39)	1638 (36.80)	1452 (36.35)	
2	2555 (13.19)	690 (13.91)	730 (14.51)		3069 (14.68)	634 (14.24)	582 (14.57)	
≥3	1056 (5.45)	272 (5.48)	336 (6.68)		1049 (5.02)	246 (5.53)	243 (6.08)	
Total	19,372 (65.97)	4962 (16.90)	5030 (17.13)		20,903(71.22)	4451 (15.17)	3994 (13.61)	

*P*-values were obtained by chi-square test

After adjusting for confounding variables (age, gender, residence, per capita household income, and physical activity), the risks of abdominal obesity, high TG, low HDL-C, and MetS were higher in high-level sedentary time group than those in low-level sedentary time group (OR 1.41, 95% CI 1.31–1.52, *p* < 0.0001; OR 1.16, 95% CI 1.08–1.25, *p* < 0.0001; OR 1.12, 95% CI 1.03–1.22, *p* < 0.01; OR 1.35, 95% CI 1.20–1.52, *p* < 0.0001, respectively) (Table 4). In boys, compared with low-level sedentary time group, the risks of abdominal obesity, high TG, low HDL-C, and MetS were higher in high-level sedentary time group (OR 1.41, 95% CI 1.27–1.56, *p* < 0.0001; OR 1.18, 95% CI 1.06–1.32, *p* < 0.01; OR 1.24, 95% CI 1.10–1.40, *p* < 0.01; OR 1.32, 95% CI 1.12–1.55, *p* < 0.01, respectively) (Table 4). In girls, the risks of abdominal obesity, high TG, and MetS were higher for those with high level of sedentary behavior (OR 1.42, 95% CI 1.28–1.57, *p* < 0.0001; OR 1.14, 95% CI 1.04–1.26, *p* < 0.01; OR 1.37, 95% CI 1.16–1.63, *p* < 0.01, respectively) (Table 4).

In Table 5, after adjusting for confounding variables (age, gender, residence, per capita household income, and physical activity), the risks of abdominal obesity and MetS were 1.15 and 1.14 times higher for those who reported ≥ 3 h/day of screen time, respectively (OR 1.15, 95% CI 1.08–1.22, *p* < 0.01; OR 1.14, 95% CI 1.03–1.25, *p* < 0.01, respectively).

**Table 4:** Associated factors of the sedentary time and MetS and its components using logistic regression analysis (OR (95% CI of OR) )

Variables	Sedentary time	Abdominal obesity	High TG	Low HDL-C	Hyperglycemia	Hypertension	MetS
Multivariate ^a^	Low level	1.00	1.00	1.00	1.00	1.00	1.00
	Medium level	1.20 (1.13,1.27)***	1.09 (1.03,1.15)**	1.08 (1.00,1.15)*	1.02 (0.86,1.21)	0.97 (0.93,1.01)	1.23 (1.12,1.35)***
	High level	1.41 (1.31,1.52)***	1.16 (1.08,1.25)***	1.12 (1.03,1.22)**	1.13 (0.92,1.38)	0.93 (0.88,0.98)*	1.35 (1.20,1.52)***
Boys ^b^	Low level	1.00	1.00	1.00	1.00	1.00	1.00
	Medium level	1.14 (1.05,1.24)**	1.03 (0.94,1.12)	1.13 (1.02,1.25)*	0.93 (0.76,1.15)	0.93 (0.87,0.98)**	1.08 (0.94,1.23)
	High level	1.41 (1.27,1.56)***	1.18 (1.06,1.32)**	1.24 (1.10,1.40)**	1.05 (0.81,1.35)	0.90 (0.83,0.97)**	1.32 (1.12,1.55)**
Girls ^b^	Low level	1.00	1.00	1.00	1.00	1.00	1.00
	Medium level	1.26 (1.16,1.37)***	1.14 (1.06,1.23)**	1.04 (0.95,1.15)	1.20 (0.90,1.59)	1.01 (0.95,1.07)	1.41 (1.23,1.62)***
	High level	1.42 (1.28,1.57)***	1.14 (1.04,1.26)**	1.02 (0.90,1.15)	1.29 (0.92,1.82)	0.97 (0.90,1.05)	1.37 (1.16,1.63)**

a: adjusted for age, gender, residence, per capita household income, and physical activity

b: adjusted for age, residence, per capita household income, and physical activity

*: *p* < 0.05, **: *p* < 0.01, ***: *p* < 0.001

**Table 5:** Associated factors of the screen time and MetS and its components using logistic regression analysis (OR (95% CI of OR) )

Variables	Screen time	Abdominal obesity	High TG	Low HDL-C	Hyperglycemia	Hypertension	MetS
Multivariate ^a^	< 2 h/day	1.00	1.00	1.00	1.00	1.00	1.00
	2–3 h/day	1.03 (0.97,1.10)	0.95 (0.89,1.01)	0.91 (0.85,0.98)*	0.96 (0.80,1.14)	0.99 (0.95,1.04)	1.02 (0.92,1.13)
	≥ 3 h/day	1.15 (1.08,1.22)***	1.06 (0.99,1.13)	1.02 (0.95,1.09)	1.03 (0.86,1.22)	0.93 (0.89,0.98)**	1.14 (1.03,1.25)**
Boys ^b^	< 2 h/day	1.00	1.00	1.00	1.00	1.00	1.00
	2–3 h/day	0.99 (0.90,1.08)	0.98 (0.90,1.08)	0.89 (0.80,0.98)*	0.93 (0.75,1.17)	0.97 (0.91,1.03)	0.93 (0.81,1.07)
	≥ 3 h/day	1.08 (0.99,1.17)	1.07 (0.98,1.17)	1.02 (0.93,1.12)	0.96 (0.77,1.19)	0.94 (0.88,1.00)*	1.06 (0.93,1.21)
Girls ^b^	< 2 h/day	1.00	1.00	1.00	1.00	1.00	1.00
	2–3 h/day	1.08 (0.99,1.19)	0.92 (0.85,1.01)	0.93 (0.84,1.04)	1.00 (0.74,1.35)	1.02 (0.95,1.09)	1.12 (0.97,1.29)
	≥ 3 h/day	1.24 (1.14,1.36)***	1.05 (0.96,1.14)	0.99 (0.88,1.10)	1.18 (0.88,1.58)	0.92 (0.86,0.99)*	1.22 (1.05,1.41)**

a: adjusted for age, gender, residence, per capita household income, and physical activity

b: adjusted for age, residence, per capita household income, and physical activity

*: *p* < 0.05, **: *p* < 0.01, ***: *p* < 0.001

## Discussion

According to the Report on Chinese Residents’ Chronic Diseases and Nutrition 2020, the physical activity deficiency (Moderate-to-vigorous intensity physical activity < 60 min/day) rate among Chinese children and adolescents aged 6–17 was 86.0% [28]. The most recent global data show that 81% of adolescents does not meet WHO’s recommendation levels of physical activity [29]. Physical inactivity and sedentary behavior of children and adolescents drew extensive attention worldwide.

The present study focused on the associations between hot problems: sedentary behavior, screen time and MetS. It showed that the overall prevalence of MetS among 7-17-year-old Chinese children and adolescents was 5.45%, which was higher than 2.4% in Beijing [30] and Guangzhou [31]. This could be explained by the differences in age range and diverse criteria of MetS that were applied, and most importantly, the different regions in which we studied. To our knowledge, this is the first nationwide study that discover the association between sedentary behavior, screen time and MetS among students. Although some studies reported no sex difference in the prevalence of MetS [32, 33], our study found that boys were more likely to have MetS than girls, which was consistent with most studies [22, 30, 31]. Except differences in physiological and genetic structure, it remains challenging to find the potential mechanisms responsible for gender disparities. Interestingly, our previous study showed that screen time of Chinese boys were higher than that of girls [34]. Starting from this aspect, a deeper exploration could be made. Moreover, we observed a positive relationship between MetS and age and this is congruent with previous studies [30, 35]. Possible reasons include changes in hormone regulation of appetite and satiety and fat distribution during puberty [36].

This study found that in boys, the prevalence of abdominal obesity, high TG, low HDL-C, and MetS was higher in high-level sedentary time group; while in girls, the prevalence of abdominal obesity, high TG, hyperglycemia, and MetS was higher in those with high-level sedentary behavior. This indicates that sex plays a role in the effects of sedentary behavior on MetS, which is consistent with the Beijing study [30]. The results showed a reverse relationship between the prevalence of HTN and sedentary behavior. This may be because some of the important confounders, such as diet, were not considered. Excessive sodium intake and low potassium intake, as well as a low ratio of potassium to sodium intake are significant risk factors for HTN in China [37]. Therefore, it needs to be considered with dietary factors in future studies. The result revealing that compared with low-sedentary time group, high sedentary behaviors had higher risks of MetS and its components is congruent with other studies. According to Yin et al.,high sedentary behavior time was associated with higher risks of MetS components, including abdominal obesity, high TG, and low HDL-C [30]. A longitudinal study suggested that men with medium to high level of sedentary time had 65% and 76% higher risks of having MetS, respectively [38]. A Brazilian study found that higher screen-based sedentary time was positively related to MetS among adolescents aged 12 to 17 years [39]. According to Berg et al., an extra hour of accelerometer-based sedentary behavior was associated with a 39% increase risk for MetS [40]. The present study found that the odds ratio of HTN in high-level sedentary time group was less than 1, indicating a protective effect of sedentary behavior on HTN. Nevertheless, its 95% CI of OR was close to 1, indicating a null finding. Therefore, this showed that HTN had no relationship with sedentary behavior. A cohort study also found no association between noninteractive sedentary behaviors (such as TV viewing) but not interactive sedentary (such as driving and computer use) and self-reported incident HTN [41]. This suggests that further grouping the sedentary time into different activities can make a deeper investigation and explanation of the associations in the future study.

In the present study, the risk of abdominal obesity was higher for those with high screen time. Indeed, a Brazilian study suggested that students in post puberty phase with screen time ≥ 3 h/day was 1.43 times more likely to have abdominal obesity [42]. A Korean study also found that adolescents aged 12–18 years with daily screen time ≥ 5 h had 3 times increased risk of abdominal obesity compared to those with daily screen time < 3 h [43]. One possible reasons is that adolescents with high screen time consumed less unprocessed foods and more ultra-processed foods [42]. Dietary patterns characterized by high consumption of sodium, animal fat, and refined carbohydrates, and low consumption of fiber are related to risks of elevated blood pressure and increased body adiposity in adolescents [44]. In addition to abdominal obesity, the study showed that screen time is a risk factor for MetS as well. According to Khan et al., students with screen time ≥ 2 h/day were 2.2 times more likely to have MetS [19]. A NHANES study also found a dose-dependent manner between screen time and MetS [45]. A Guangzhou study including 4523 children and adolescents aged 6–13 years suggested that compared to low-level screen time group, medium and high-level screen time groups had an increased likelihood of MetS [31].

Inconsistent with the previous study which found no association between abdominal obesity and sedentary behavior among inactive males and females [46], our study found both screen time and sedentary behavior were associated with abdominal obesity. Abdominal obesity, an establish risk factor and the most prevalent manifestation of MetS, is a maker of dysfunctional adipose tissue. A cohort study revealed that both screen-based and non-screen based sedentary behavior had associations with abdominal adipose tissue deposition [47]. And a robust inverse association was observed for physical activity and visceral adipose tissue accumulation [47]. Although our study controlled physical activity variables, it is recommended to be physically active to prevent abdominal obesity and MetS.

The strengths of this study include a large sample size that incorporated children and adolescents in 31 provinces that could represent the whole nation data, standardized questionnaires, anthropometric measurements, and clinical examinations. Nevertheless, several limitations warrant attention. First, it was a cross-sectional study which could not clarify the causal relationship between sedentary behavior, screen time and MetS. In addition, although completed face-to-face by trained investigators, all questionnaires were self-reported, which was easily subject to recall bias. Moreover, a subjective measure of self-reporting as a means of measuring the sedentary time and the screen time was not precise. Accelerometers and inclinometers that capture data directly from electronic devices are more objective and reliable [48]. However, several shortcomings including lack of a standard cutoff point, difficulty of examining patterns of sedentary time, and poor compliance with wearing the device limit the widespread use of them [48]. Maybe the future study that could combine subjective with objective methods could solve the problem. Besides, our study included both screen-based and non-screen-based sedentary behavior into sedentary behavior. Maybe we will separate them to examine the relationship between each of them and MetS in the future study.

## Conclusions

In summary, this study shows that high levels of sedentary time and screen time were associated with an increased likelihood of MetS among Chinese children and adolescents. Thus, in order to avoid MetS, students, parents and schools should work together to develop a healthy lifestyle to reduce sedentary behavior and screen time.

## Data Availability

The datasets used and analyzed during the current study are not publicly available for ethical and privacy reasons but are available from the corresponding author upon reasonable request.
